# Lesson learned from the pandemic: Isolation and hygiene measures for COVID-19 could reduce the nosocomial infection rates in oncology wards

**DOI:** 10.1177/10781552211043836

**Published:** 2021-09-30

**Authors:** Deniz C Guven, Imdat Eroglu, Rashad Ismayilov, Ege Ulusoydan, Oktay H Aktepe, Gulcin Telli Dizman, Zafer Arik, Omer Dizdar, Serhat Unal, Gokhan Metan, Neyran Kertmen

**Affiliations:** 1 Department of Medical Oncology, 37515 Hacettepe University Cancer Institute, Turkey; 2 Department of Internal Medicine, 37515 Hacettepe University, Turkey; 3 Department of Clinical Microbiology and Infectious Disease, 37515 Hacettepe University, Turkey

**Keywords:** Cancer, COVID-19, inpatient ward, nosocomial infection, pandemic

## Abstract

**Introduction:**

It was previously demonstrated that seasonal influenza incidence was significantly decreased during the COVID-19 pandemic, possibly due to respiratory and hygiene precautions. From this point, we hypothesized that the COVID-19 precautions could lead to a decrease in nosocomial infection rates in oncology inpatient wards.

**Methods:**

We evaluated the nosocomial infection rates in an inpatient palliative oncology ward in the first 3 months of the COVID-19 pandemic in our country and compared this rate with the same time frame of the previous year in our institution.

**Results:**

The percentage of nosocomial infections complicating the hospitalization episodes were significantly reduced in the first 3 months of the pandemic compared to the previous year (43 vs. 55 nosocomial infection episodes; 18.6% vs. 32.2%, *p* = 0.002). The decrease in the nosocomial infections was consistent in the different types of infections, namely pneumonia (4.8% vs. 7.6%), urinary tract infection (5.2% vs. 7.6%), bacteremia (5.2% vs. 7%) and intraabdominal infections (2.6% vs. 3.5%). The median monthly disinfectant use was significantly increased to 98 liters (interquartile range: 82 – 114) in 2020 compared to 72 L (interquartile range: 36 – 72) in 2019 (*p* = 0.046).

**Conclusion:**

The continuation of the simple and feasible hygiene and distancing measures for healthcare workers and patient relatives and adaptations for earlier discharge could be beneficial for preventing nosocomial infections in oncology wards. These measures could be implemented routinely even after the COVID-19 pandemic for patient safety, especially in settings with higher nosocomial infection rates like inpatients palliative care units.

## Introduction

Coronavirus disease 2019 (COVID-19) created the most significant pandemic in a century and changed the World in the last year.^1^ Nosocomial contamination is one of the most critical transmission routes of infection and it was suggested up to one-third of COVID-19 cases could be nosocomial.^2^ Patients with cancer are among the most susceptible populations affected by the COVID-19 pandemic with increased hospitalization, mechanical ventilation, and death rates,^3,4^ so preventing nosocomial COVID-19 contamination is paramount.

Due to prevent nosocomial COVID-19 contamination, several isolation and hygiene measures were implemented in the oncology hospitals. These measures included,^5,6^

- placement of warning signs for social distancing,
- the use of masks by the healthcare workers,
- implementation of triage area for the body temperature measurements and symptom questioning at the entrance of the hospital,
- increased use of telemedicine,
- restrictions of the inpatient visits,
- efforts for a shorter hospital stay, and
- hospitalization of patients with respiratory symptoms in isolated rooms.

It was previously demonstrated that seasonal influenza incidence was significantly decreased during the COVID-19 pandemic,^7^ possibly due to respiratory and hygiene precautions for COVID-19. From this point, we hypothesized that the COVID-19 precautions could lead to a decrease in nosocomial infection rates in oncology inpatient wards. To test this hypothesis, we evaluated the nosocomial infection rates in an inpatient palliative oncology ward in the first 3 months of the COVID-19 pandemic between 11 March 2020 and 10 June 2020 in our country^8^ and compared this rate with the same time frame of the previous year in our institution.

## Methods

### Structure of inpatient ward

The study was conducted in Hacettepe University Oncology Hospital, an ESMO designated cancer center, which was served as a clean hospital during the pandemic. The inpatient ward consisted of fifteen rooms with two beds per room. Regular patient visits were conducted twice a day by a medical oncologist for supervision and treatment planning. A medical oncology resident was present in the ward during working hours and was on-call duty during the night shifts. Internal medicine residents were present during the daytime and night shifts. Additionally, daily visits to the inpatient ward were conducted by infectious disease physicians. Three nurses and two staff from housekeeping services worked for every eight-hour shift. During the COVID-19 pandemic, all staff were regularly educated for COVID-19 and additional warning cards were placed for patients with respiratory symptoms or the suspicion of COVID-19.

### Nosocomial infection definitions and statistical analysis

Nosocomial infection events were defined as the infections developed after the 48 h of hospitalization, and the infections developed in the following 72 h after discharge as suggested in the literature.^9,10^ All patients who were hospitalized at least for 48 h were included in the study. The patients hospitalized for imaging and one-day chemotherapy discharged on the same day of hospitalization were excluded. The types of infections such as pneumonia, urinary tract infection, intraabdominal infection, and bacteremia were identified according to the infectious disease consultation notes or follow-up records. Additionally, total hand disinfectant uses were recorded for 2 years.

The descriptive features were expressed by the medians, interquartile ranges (IQR), and percentages where ever appropriate. The baseline characteristics of patients were compared with *T*-test and chi-square tests. The rate of nosocomial infections during hospitalization episodes was compared with the chi-square and Fischer-exact tests. The median monthly disinfectant uses were compared with Mann-Whitney *U*-test. All statistical analyses were performed in SPSS 25 (IBM Inc., Armonk, NY, USA) software, and a type-I error level of 5% (*p* < 0.05) was considered as the threshold limit for statistical significance. All the performed procedures in the present study complied with the 1964 Helsinki declaration and its later amendments. This study was approved by Hacettepe University Ethics Committee.

## Results

The data of 231 patients from 2020 and 171 patients from 2019 were retrospectively evaluated. The median ages of the patients (61 (IQR 49–68) vs. 62 (IQR 52–68) (*p* = 0.840) for 2020 and 2019, respectively), and median duration of hospitalization were similar for 2 years (9 (IQR 3–18) vs. 8 (IQR 5–17) (*p* = 0.840) for 2020 and 2019, respectively). More female patients were hospitalized in 2020 (50.6% vs. 39.2%, *p* = 0.023). During the first 3 months of the pandemic, six patients had COVID-19 suspicion in chest scans and 92 patients had either fever or respiratory symptoms. A total of 98 SARS-CoV-2 polymerase chain reactions (PCRs) were conducted, and results of all PCR tests were negative.

**Figure 1.**
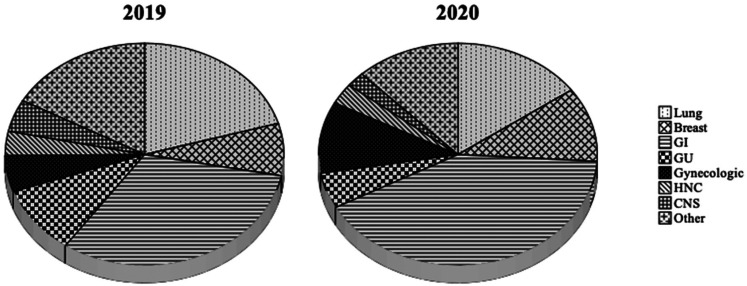
Distribution of the primary tumors in 2019 and 2020.

Lung cancer was the most common diagnosis (35/171) in 2019, followed by the pancreas (15/171) and breast cancer (13/171). The primary tumor distribution was somewhat different in between 2 years, with colorectal cancer being the most frequent primary (36/231) and followed by lung cancer (35/231) and breast cancer (25/231). The gastrointestinal (GI) tumors as a group were the most frequent primary tumor in the study population (31.6% in 2019 and 41.1% in 2020) (Figure 1). Most of the patients had advanced stage disease (93% in 2019 and 88.3% in 2020).

The percentage of nosocomial infections complicating the hospitalization episodes were significantly reduced in the first 3 months of the pandemic compared to the previous year (43 vs. 55 nosocomial infection episodes; 18.6% vs. 32.2%, *p* = 0.002). The decrease in the nosocomial infections was consistent in the different types of infections, namely pneumonia (4.8% vs. 7.6%), urinary tract infection (5.2% vs. 7.6%), bacteremia (5.2% vs. 7%), and intraabdominal infections (2.6% vs. 3.5%). However, the utilization rates of carbapenems (*p* = 0.204), colistin (*p* = 0.104), vancomycin, or teicoplanin (*p* = 0.318) were similar between 2 years (Table 1). The rate of nosocomial infections caused by multidrug-resistant bacteria was similar between 2 years (*p* = 0.677) (Table 2). The median monthly disinfectant use was significantly increased to 98 L (IQR 82–114) in 2020 compared to 72 L (IQR 36–72) in 2019 (*p* = 0.046).

**Table 1.** The percentage of patients with nosocomial infections or wide spectrum antibiotic usage during hospitalization in 2019 and 2020.

Year		2019	2020	*p*-value
		*n* (%)	*n* (%)	
Nosocomial infection	Absent	116 (67.8)	188 (81.4)	0.002
	Present	55 (32.2)	43 (18.6)	
Bacteremia	Absent	159 (93.0)	219 (94.8)	0.446
	Present	12 (7.0)	12 (5.2)	
Diarrhea	Absent	169 (98.8)	229 (99.1)	> 0.99
	Present	2 (1.2)	2 (0.9)	
Intraabdominal infection	Absent	165 (96.5)	225 (97.4)	0.596
	Present	6 (3.5)	6 (2.6)	
Pneumonia	Absent	158 (92.4)	220 (95.2)	0.235
	Present	13 (7.6)	11 (4.8)	
Urinary tract infection	Absent	158 (92.4)	219 (94.8)	0.323
	Present	13 (7.6)	12 (5.2)	
Carbapenem	Absent	142 (83.0)	180 (77.9)	0.204
	Present	29 (17.0)	51 (22.1)	
Colistin	Absent	168 (98.2)	220 (95.2)	0.104
	Present	3 (1.8)	11 (4.8)	
Vancomycin/Teicoplanin	Absent	153 (89.5)	199 (86.1)	0.318
	Present	18 (10.5)	32 (13.9)	

**Table 2.** The isolation rate of multidrug-resistant bacteria from nosocomial infections in 2019 and 2020.

Year	2019 (*n*)	2020 (*n*)
Methicillin-resistant *Staphylococcus epidermidis* (MRSE)	2	0
Methicillin-resistant *Staphylococcus aureus* (MRSA)	0	2
Extended-spectrum beta-lactamase-producing *Escherichia coli* or *Klebsiella* species	12	12
Carbapenem resistant *Klebsiella pneumoniae*	1	1
*Stenotrophomonas maltophilia*	1	0

## Discussion

Herein, we demonstrated a significant decrease in the rate of nosocomial infections in an inpatient oncology ward during the first 3 months of the pandemic. The decrease was consistent in almost all infection types. We think that the preventive measures for COVID-19, led by improved hand hygiene, could be the reason for this decrease.

Infections are among the most important causes of morbidity in patients with cancer^11,12^ and frequently complicates the course of hospitalization,^13^ as evident in our study demonstrating more than 15% nosocomial infection rate during hospitalization. Our nosocomial infection rates were somewhat higher than two recent comprehensive studies reporting lower nosocomial infection rates (6.9% and 15.5%).^14,15^ The possible reasons for this difference could be the exclusion of patients discharged on the same day of hospitalization and our study setting (the palliative care ward). Patients hospitalized in the palliative care wards tend to be more fragile, have frequent healthcare contact, and have recurrent hospitalizations, which makes them vulnerable to nosocomial infections.^16,17^

International Societies recommend several precautions and infection control measures, and the hospital infection control units implement these recommendations to control nosocomial infections.^12^ Hand hygiene is one of the most important nosocomial infection prevention interventions,^18^ although the compliance rates are very variable and generally far below the desired rates.^19,20^ However, compliance to hand hygiene is expected to be improved due to the significant emphasis on this issue in the media and regular COVID-19 education emphasizing the hand hygiene during the pandemic in most institutions. Similarly, hand sanitizer uses are expected to increase during the COVID-19 pandemic as seen in our experience. All these factors could lead to optimized hand hygiene during the pandemic and could be the main reason for the decreased nosocomial infection rates in our study. Although we could not be able to evaluate the difference due to no PCR-confirmed influenza cases during our study frame in both years, social distancing, use of masks and the prompt respiratory isolation could lead to decreased rates of nosocomial influenza and other respiratory virus contaminations.

Our study has several limitations. The study was a single-center retrospective study, including a modest number of cases. Our institution served as a clean hospital during the pandemic, which impairs the generalizability of our results to centers that are overwhelmed by a high number of cases. However, the clean hospital setting could be a better reflector of the precautions' efficacy without the confounding of nosocomial COVID-19 contamination. Additionally, we categorized the types of infections based on infectious diseases consultations rather than the Center for Disease Control and Prevention criteria which is commonly used in the epidemiological investigations. Lastly, our study was conducted during a period of strict precautions but no lockdown. This limits our results’ generalizability to countries that did not adopt strict measures like Sweden and countries that applied a nationwide lockdown like New Zealand during the pandemic. Despite these limitations, we could give a general perspective about the off-target benefits of hygiene precautions for COVID-19 on nosocomial infections.

In conclusion, we observed a significant decrease in the nosocomial infection rates during the COVID-19 pandemic in our institution. The continuation of the simple and feasible hygiene and distancing measures for healthcare workers and patient relatives and adaptations for earlier discharge could be beneficial for preventing nosocomial infections in oncology wards. These measures could be implemented routinely even after the COVID-19 pandemic for patient safety, especially in settings with higher nosocomial infection rates like inpatient palliative care units.

## Compliance with ethical standards

All procedures performed in studies involving human participants were in accordance with the ethical standards of the institutional and/or national research committee and with the 1964 Helsinki declaration and its later amendments or comparable ethical standards. The study was approved by the ethics committee of Hacettepe University and the Turkish Ministry of Health.
